# Genome Sequence and Phylogeny of a *Bean Yellow Mosaic Virus* Isolate Obtained from a 14-Year-Old Australian Lentil Sample

**DOI:** 10.1128/MRA.01437-19

**Published:** 2020-01-09

**Authors:** S. Maina, L. Zheng, S. King, M. Aftab, N. Nancarrow, P. Trębicki, B. Rodoni

**Affiliations:** a Agriculture Victoria Research, Horsham, VIC, Australia; b Agriculture Victoria Research, AgriBio, Bundoora, VIC, Australia; Portland State University

## Abstract

Using RNA strand-specific sequencing followed by *de novo* assembly, a *Bean yellow mosaic virus* (BYMV) genome was obtained from a lentil sample (Aus14BY) collected in Victoria, Australia, in 2005. When compared with 51 BYMV genomes, it closely resembled the Western Australian isolate PN83A (Lupinus angustifolius), with 98.4% nucleotide identity.

## ANNOUNCEMENT

*Bean yellow mosaic virus* (BYMV) (family *Potyviridae*, genus *Potyvirus*) is a single-stranded positive-sense RNA virus that occurs globally. It has a wide host range that includes monocots and dicots in both domesticated and wild plant species (1, 2). It is transmitted nonpersistently by several aphid species (2). Seed transmission occurs at a low level, which can lead to inadvertent introduction of the virus into new field sites (2, 3). BYMV causes serious disease and losses in many cultivated crops worldwide, and early infection results in systemic necrosis, leading to plant death (4, 5). In Australia, BYMV infects most temperate pulses, including faba beans (Vicia faba), field peas (Pisum sativum), lentils (Lens culinaris), and lupins (*Lupinus* spp.). As part of a project that aims to apply high-throughput sequencing (HTS) to improve the efficiency of pulse seed diagnostic testing, one leaf sample (preserved with CaCl_2_) that had been collected from a lentil crop growing in Wimmera (Victoria, Australia) and showing virus-like symptoms was subjected to HTS for virus detection, and a complete BYMV (14BY) genome was obtained.

Total RNA was extracted from the sample using a Zymo Research plant RNA miniprep kit, and quality control checks were performed as described previously (6). The RNA sequencing library was prepared using the TruSeq stranded total RNA sample preparation kit with Ribo-Zero Plant (Illumina, San Diego, CA), as described previously (7, 8). The library was diluted, denatured, and then sequenced using a MiSeq v3 kit (Illumina) with paired-end reads (2 by 251 cycles).

Quality control analysis of the fastq files was performed using Trim Galore (9), with the minimum sequence length set to 50 bp and the minimum required adapter overlap (stringency) set to 1 bp. The *de novo* assembly was performed using the metaSPAdes v3.13.0 genome assembler (10) with default settings. The contigs of interest were imported into Geneious (11), multiple alignment with GenBank sequences was performed using MUSCLE (12), and annotation was performed with transfer annotation selected and similarity set at 90%, while other settings were left as defaults (11). The alignment of 51 BYMV genomes was used to infer a maximum-likelihood phylogenetic tree using MEGA 7.0.14 (13). The tree compared the sequence of the BYMV lentil isolate with other Australian BYMV sequences (14) and all other worldwide BYMV sequences that were available in GenBank.

**FIG 1:**
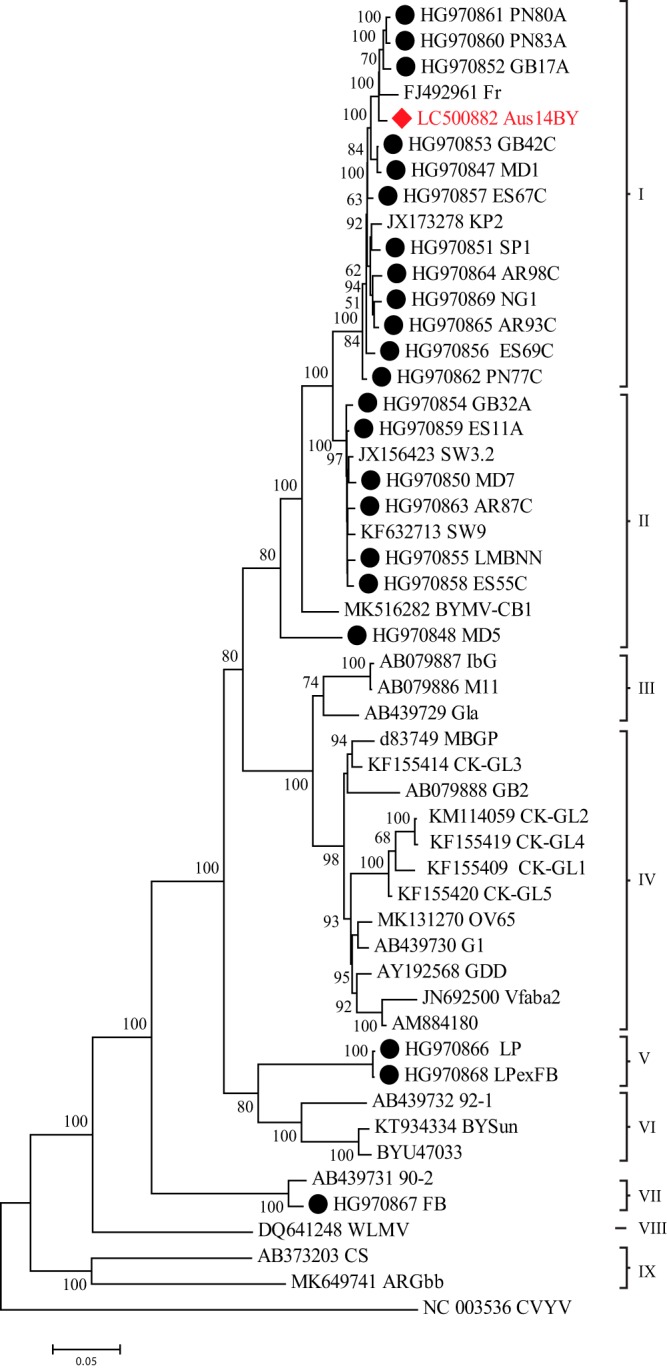
Alignment of BYMV genomes. The 51 BYMV genomes were aligned using MUSCLE (12) by setting the number of iterations to 10 and the rest of the settings to defaults. The evolutionary history was inferred with the maximum-likelihood tree using the Tamura-Nei model. The codon positions included were first plus second plus third plus noncoding, and all ambiguous nucleotide positions were removed for each sequence. The tree was created using MEGA 7.0.14 (13) with 1,000 replicates. Bootstrap values are percentages, with values over 50% shown at the nodes. The tree was rooted using *Clover yellow vein virus*. Black circles, previously published Australian sequences; red diamond, newly sequenced isolate.

The run yielded 3,588,734 raw reads, and 3,541,904 reads remained after quality control procedures. *De novo* assembly generated 12,171 contigs. All of the 12,171 contigs were subjected to a BLASTN search using BLAST+ v2.7 (15) to check for hit matches to BYMV. Only 1 contig was highly similar to BYMV, with 98.4% nucleotide identity, and it closely resembled the Western Australian BYMV isolate PN83A (Lupinus angustifolius). This contig consisted of 9,868 nucleotides, with an average coverage depth of 2,307× and a GC content of 39.4%. The phylogenetic analysis revealed nine minor phylogroups (phylogroups I to IX) (Fig. 1), representing original host isolates (dicot and monocot), as described by Kehoe et al. (14). Isolate Aus14BY fit in minor phylogroup I (bootstrap support value, 100%) (Fig. 1), which included predominantly monocot species that had been previously sequenced from Australia. Isolates CS and ARGbb (minor phylogroup IX) grouped separately from the other eight phylogroups, which might reflect recombination, but a further recombination study would be required to evaluate this hypothesis.

This study forms part of a wider safer-seed certification project, which aims to apply HTS to increase the efficiency of current biosecurity procedures to safeguard the movement of pulse germplasm into and out of Australia. This new sequence from lentil also provides new phylogenetic insights into BYMV.

### Data availability.

The sequence described here was deposited in GenBank under accession number LC500882. The raw reads were deposited in GenBank under SRA number SRR10420664, which is part of BioProject number PRJNA588304.
